# Case report: Exploring Lynch Syndrome through genomic analysis in a mestizo Ecuadorian patient and his brother

**DOI:** 10.3389/fmed.2024.1498290

**Published:** 2024-12-17

**Authors:** Patricia Guevara-Ramírez, Viviana A. Ruiz-Pozo, Santiago Cadena-Ullauri, Elius Paz-Cruz, Rafael Tamayo-Trujillo, Aníbal Gaviria, Francisco Cevallos, Ana Karina Zambrano

**Affiliations:** ^1^Centro de Investigación Genética y Genómica, Facultad de Ciencias de la Salud Eugenio Espejo, Universidad UTE, Quito, Ecuador; ^2^Hemocentro Nacional, Cruz Roja Ecuatoriana, Quito, Ecuador

**Keywords:** lynch syndrome, colorectal cancer, genomic, ancestral, Ecuadorian

## Abstract

Lynch Syndrome (LS) is a hereditary disorder characterized by genetic mutations in DNA mismatch repair genes, affecting approximately 0.35% of the population. LS primarily increases the risk of colorectal cancer (CRC), as well as various other cancer types like endometrial, breast, and gastric cancers. Microsatellite instability, caused by MMR gene mutations, is a key feature of LS, impacting genes such as *MLH1*, *MSH2*, *MSH6*, and *PMS2*. Pathology tests studying microsatellite instability and immunohistochemical staining are used to diagnose LS. Furthermore, next-generation sequencing (NGS) allows for a thorough investigation of cancer susceptibility genes. This approach is crucial for identifying affected individuals and managing their care effectively. This study evaluated two siblings who harbored a mutation in the *MLH1* gene associated with LS. The older brother was diagnosed with CRC at 24, while the younger brother remains asymptomatic at 7 years old. Genetic testing confirmed the presence of the *MLH1* mutation in both siblings. Ancestry analysis showed a mix of African, European, and Native American heritage, common among Ecuadorians. Both siblings shared a family history of cancer, suggesting hereditary factors. Treatment involved surgery and chemotherapy for the older brother, emphasizing the importance of genetic testing for siblings with a cancer family history. NGS plays a pivotal role in identifying genetic mutations and guiding treatment decisions, demonstrating its significance in managing LS and other hereditary cancers.

## Introduction

Lynch Syndrome (LS) is an autosomal dominant inherited disorder characterized by germline pathogenic variants in DNA mismatch repair (MMR) genes. LS carriers account for 0.35% of the general population (1). Furthermore, the lifetime risk of developing cancer in individuals LS varies depending on the mutated MMR gene, with estimates of 80% for high penetrance genes such as *MLH1* and *MSH2* (2). Individuals with LS have an elevated risk of various cancers, primarily colorectal cancer (CRC) (up to 80%), as well as other cancers, including endometrial cancer (approximately 60%) (3, 4).

Colorectal cancer is the third most frequent type of cancer worldwide (5). LS represents the predominant hereditary form of CRC. Individuals with LS have an estimated cumulative lifetime risk of developing CRC of up to 52.2% in women and 68.7% in men (6). Approximately 15% of all CRCs exhibit a MMR-deficient phenotype, resulting in microsatellite instability (MSI) and lack of expression of MMR proteins (7, 8). Microsatellite instability is characterized by the accumulation of abnormal lengths of tandemly repeated mono- or dinucleotide sequences, which are caused by mutations in one or both alleles of MMR genes.

The primary MMR genes are *MLH1*, *MSH2*, *MSH6*, or *PMS2* and their function involves detecting and repairing DNA mismatches generated during DNA replication (9). The incidence of CRC varies based on the specific MMR gene mutation and the implementation of surveillance colonoscopies. Notably, higher cumulative incidence rates have been reported for individuals with mutations in *MLH1* (36–52%) and *MSH2* (30–50%) under surveillance, compared to those with mutations in *MSH6* (10–17%) and *PMS2* (3–11%) (10).

The diagnosis of LS typically relies on a combination of pathology tests including MSI testing and immunohistochemical staining. Furthermore, next-generation sequencing (NGS) has complemented this process by enabling simultaneous analysis of multiple cancer susceptibility genes through multiplex panels. This approach provides a cost-effective alternative compared to single-gene testing (11). Molecular testing, especially for LS, has garnered attention due to its ability to precisely identify affected individuals and its evolving significance in managing prognostic and therapeutic interventions (12, 13).

This study presents a case involving two young siblings from Ecuador, both harboring a mutation in the *MLH1* gene associated with LS. The older brother, aged 24 years, has been diagnosed with CRC, while the 7-year-old brother remains asymptomatic. Our study aims to highlight the role of NGS as a fundamental tool in the management and monitoring of cancer patients and their families.

## Case presentation

This report presents a case involving two brothers, a 26-year-old man (Individual A) and an 8-year-old boy (Individual B), both of whom have a significant family history of cancer.

### Individual A

**Figure 1:**
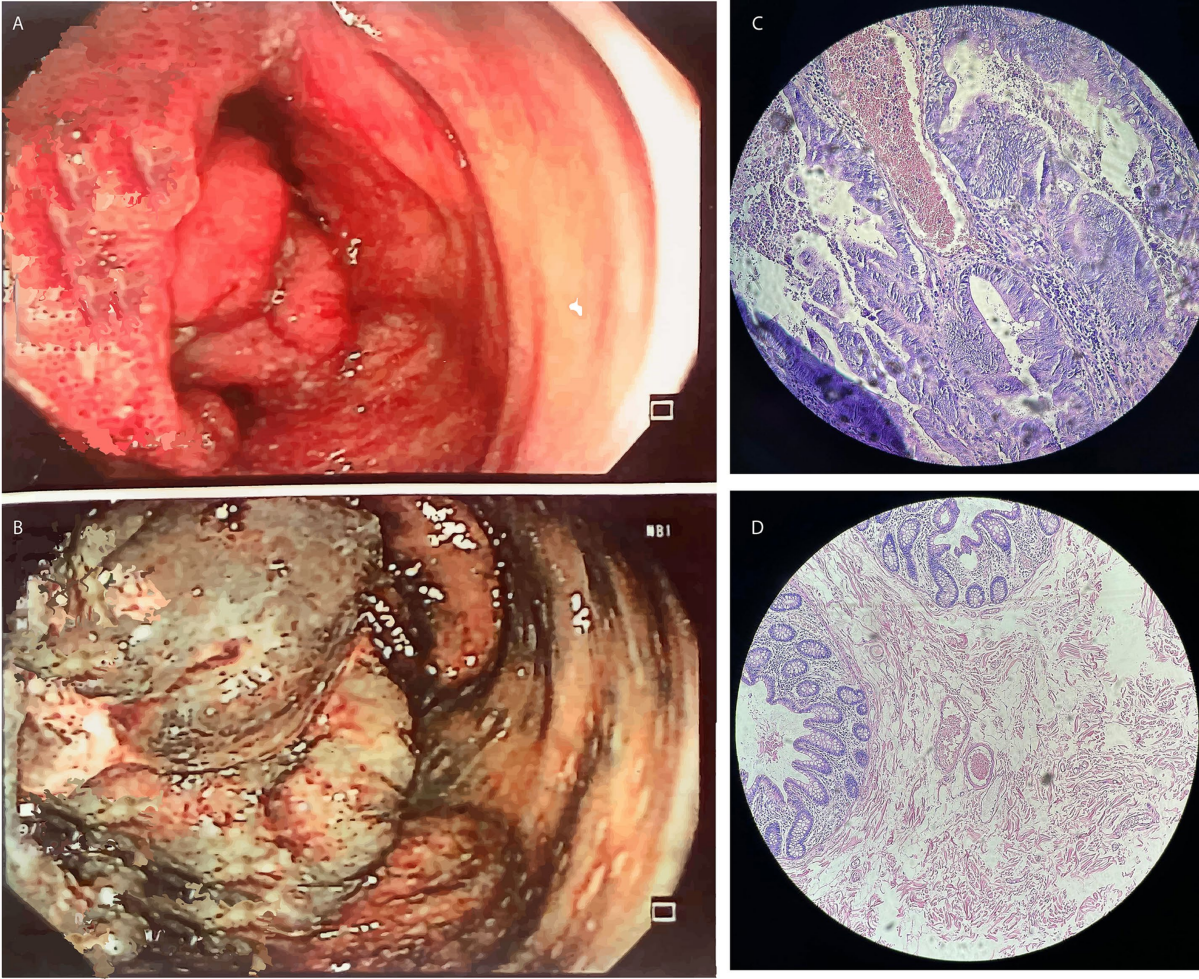
The results of the colonoscopy performed on Individual A indicated **(A,B)** Tumor mass in the cecum; **(C,D)** Hematoxylin and eosin-stained slides showing adenocarcinoma of the colon mucosa in Individual A.

Individual A reported alterations in his intestinal habits, muscle pain, and spots on the face. Hematological analysis and biochemical parameters revealed hemoglobin in the stool and anemia. A subsequent colonoscopy identified a tumor mass in the cecum, measuring approximately 6 cm. The tumor was partially obstructing the lumen of the cecum, with evidence of ulcers (Figure 1). Furthermore, a gastric biopsy revealed a well-differentiated tubulovillous adenocarcinoma of the colon. Lymph nodes were positive for malignancy, indicating lymphatic spread of the disease. The presumptive diagnosis was CRC. Genomic analyses were performed to confirm the diagnosis and assess the genetic predispositions, including LS.

### Individual B

Individual B did not exhibit any symptoms. However, due to a family history of cancer, the parents authorized genomic testing to investigate potential mutations that could affect his health in the future.

### Next generation sequencing (NGS)

The NGS analyses were conducted at the Centro de Investigación de Genética y Genómica (CIGG), Universidad UTE. Individual A and the legal guardians of individual B provided their informed consent before the process.

DNA extraction was performed from peripheral blood samples taken from individuals A and B, using the PureLink™ genomic DNA mini kit (Invitrogen, USA). In the case of Individual A, DNA was extracted from tissue samples in paraffin-embedded blocks with the same kit (PureLink™ genomic DNA mini kit).

The DNA concentrations were obtained using the Qubit™ fluorometer with the 1X dsDNA high-sensitivity (HS) and broad-range (BR) assay kits. NGS was performed on MiSeq platform (Illumina, USA), using the TruSight™ Cancer sequencing panel (Illumina, USA), which includes 255 kb and 94 genes related to different types of cancer. The bioinformatics analyses used were DRAGEN Enrichment v3.9.5, Annotation Engine v3.15, PolyPhen, Sift and Variant Interpreter v2.16.1.300.

### Ancestry analysis

For ancestry analysis, a multiplex polymerase chain reaction (PCR) of 46 ancestry-informative INDEL markers (AIMs) was conducted, following the protocol of Zambrano et al. (2019) (14). Fragment detection was performed using Genetic Analyzer 3,500 (Applied Biosystems, USA) equipment. Data Collection v3.3 and Gene Mapper v.5 software were used for data collection and analysis, respectively. The STRUCTURE v.2.3.4 software was used for ancestry study.

## Results

In individual A, genomic analyses identified a missense mutation in exon 4/19 (NM_000249.3:c.350C > T) of the *MLH1* gene and a Frameshift insertion–deletion (InDel) mutation in exon 8/9 (NM_000314.6c.968dup) of the *PTEN* gene, these mutations were detected in the paraffin-embedded tissue sample. In addition, the same *MLH1* gene mutation was observed in the DNA analysis from a blood sample. The Variant Interpreter Platform (Illumina) classified these mutations as Pathogenic for both the *MLH1* and *PTEN* genes. These analyses suggest a diagnosis consistent with LS, attributed to the variant in the *MLH1* gene.

For Individual B, who is asymptomatic and appears healthy, genomic testing revealed the presence of the same *MLH1* mutation as individual A (NM_000249.3:c.350C > T) and another mutation in the *FH* gene (NM_000143.3:c.580G > A). The latter mutation is classified as a variant of uncertain significance (VUS) and it is noteworthy because mutations in the *FH* gene have been implicated in predisposition to other cancer types (Table 1).

**Table 1:** Genetic variants identified using TruSight™ Cancer sequencing panel.

Gene	Chr	HGVSP DNA reference	HGVS Protein reference	Consequence	Predicted effect	dbSNP/dbVar ID	Genotype
MLH1^+*^	3	NM_000249.3 c.350C > T	NM_000249.3 p.(Thr117Met)	Missense variant	Pathogenic	rs63750781	Heterozygous
*PTEN^+^*	10	NM_000314.6 c.968dup	NM_000314.6 p.(Asn323LysfsTer2)	Frameshift Indels	Pathogenic	rs121913291	Heterozygous
FH^*^	1	NM_000143.3 c.580G > A	NM_000143.3 p.(Ala194Thr)	Missense variant	VUS	rs587782215	Heterozygous

*MLH1 v*ariant found in both subjects A and B. *PTEN* variant found in subject A. *FH* variant found in subject B. VUS, variants of uncertain significance. ^+^Variants of subject A; ^*^Variants of subject B.

Furthermore, the ancestry test revealed that individual A carried 6.6% African, 13.2% European, and 80.2% Native American ancestry proportions, while Individual B showed slightly different proportions with 5.4% African, 17.1% European, and 77.5% Native American ancestry (Supplementary Figure S1).

### Family history

**Figure 2:**
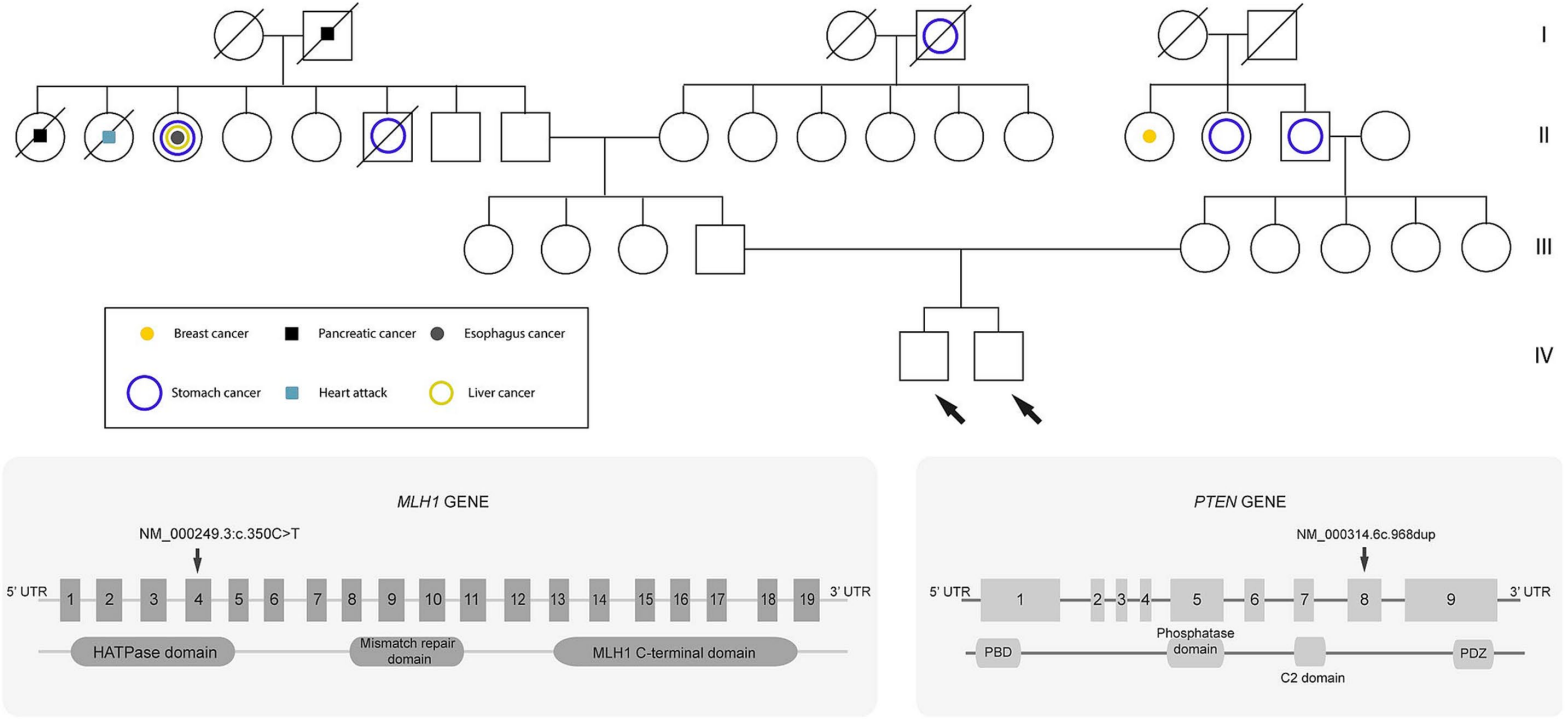
Genealogical tree of the family. Circles represent females, and squares represent males. Diagonal lines through a symbol indicate that the individual is deceased. The white circles or squares represent unaffected (healthy) individuals. Probands are indicated by the arrows.

Both siblings shared a family history of cancer. Their maternal grandfather was diagnosed with stomach cancer, and their paternal relatives had been diagnosed with pancreatic, liver, esophageal, and breast cancer. The shared genetic mutation in the *MLH1* gene between the two siblings suggests hereditary cancer (Figure 2).

### Treatment

**Figure 3:**
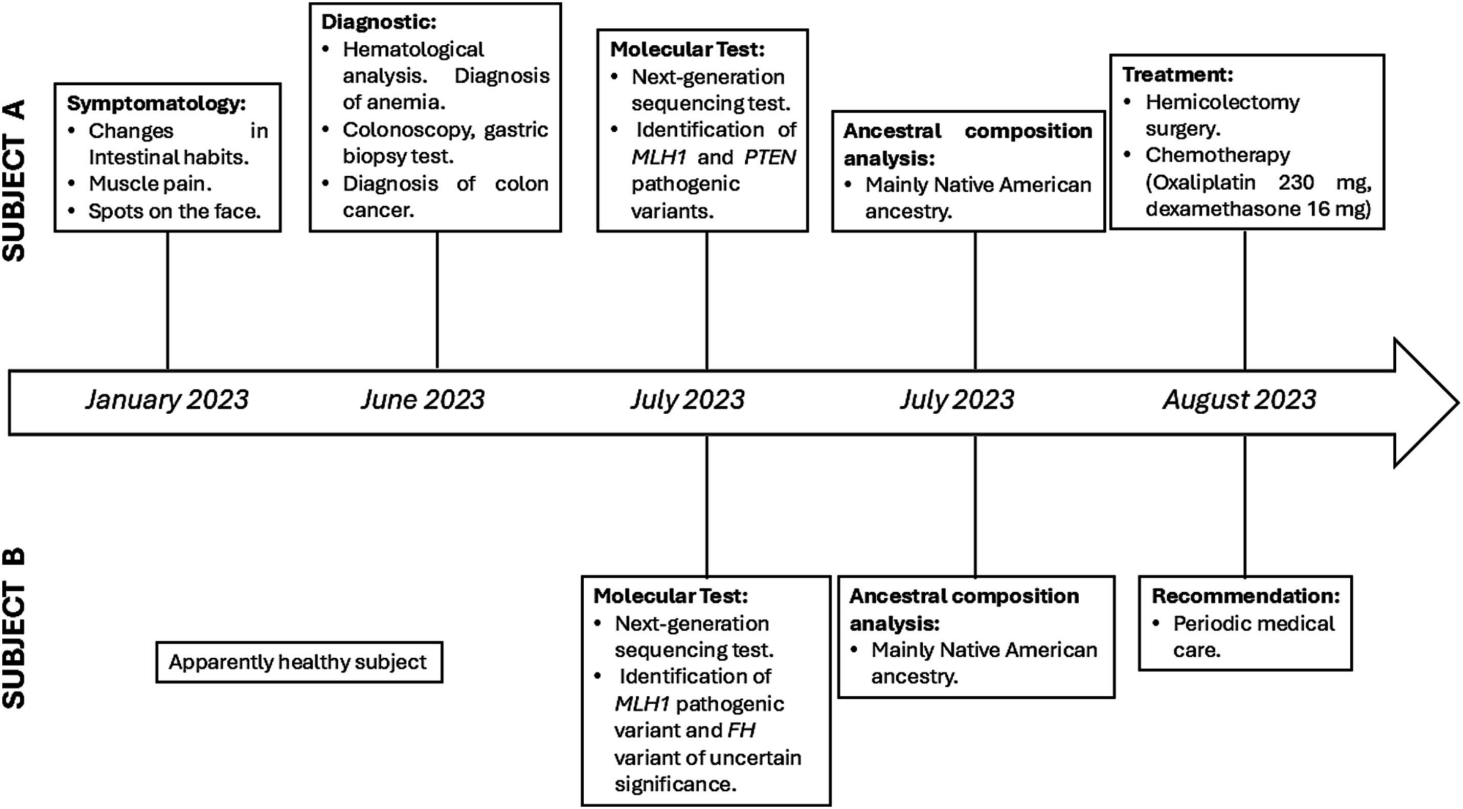
Timeline of subject A and B. The relevant episodes related to the siblings.

After diagnosis, both patients were informed about the results of the genomic test and the implications of LS. Subsequently, individual A underwent an extended right hemicolectomy and ileotransverse isoperistaltic anastomosis. Individual A was subsequently treated with chemotherapy. On the other hand, Individual B was advised to continue with follow-up medical care. This case highlights the importance of genetic testing for siblings with a family history of cancer (Figure 3).

## Discussion

Lynch Syndrome screening aims to enhance future cancer surveillance for the patient and offer germline variant testing for their at-risk relatives. However, traditional LS screening protocols, such as those based on clinical criteria like Amsterdam I and II or Bethesda guidelines, exhibit limited sensitivity, failing to detect over 40% of LS carriers (15). The advent of NGS has revolutionized clinical laboratory practices, enabling simultaneous sequencing of multiple cancer susceptibility genes with improved efficiency (11). In our investigation, we utilized NGS to analyze individuals with a familial cancer history, successfully identifying various variants, including a pathogenic mutation in the MutL Homolog 1 (*MLH1*) gene, present in two young siblings.

The *MLH1* gene encodes a protein essential for the DNA mismatch repair system forming heterodimer with the mismatch repair endonuclease PMS2, to generate MutL alpha complex. The encoded protein also participates in DNA damage signaling and can heterodimerize with MLH3 protein to form MutL gamma, involved in meiosis (16, 17). Disruption of the MLH1-PMS2 heterodimer compromises MMR, leading to the accumulation of replication errors and increased microsatellite instability. Also, disruption of the heterodimer MLH1-MLH3 could contribute to tumor growth by accumulating errors during meiosis (18–20).

LS is an autosomal dominant disorder caused by germline mutations in MMR genes, primarily *MLH1* and *MSH2*. In families with LS, over 90% of the mutations are found in these two genes (10). Mutations in the *MLH1* gene impair the protein’s ability to recognize and repair DNA damage. This leads to the accumulation of genetic errors and hinders apoptosis signaling, which could promote the development of cancer (16, 17). LS is associated with an increased predisposition to different cancer types, primarily colon cancer (occurring in 46–61% of individuals with a pathogenic *MLH1* variant) or endometrial cancer (occurring in 34–54% of individuals with a pathogenic *MLH1* variant) (2, 21). Colorectal cancer, associated with Lynch syndrome, tends to manifest at an earlier age, with an average age of diagnosis around 40 years (22).

In both subjects, the *MLH1* gene variant c.350C > T (p.Thr117Met) was identified, and notably, in Individual A, this variant was present in both blood and tissue samples. According to the InSiGHT classification, the *MLH1* variant is classified as class 5 (pathogenic) (23, 24). The c.350C > T variant affects a highly conserved domain in exon 4 of the *MLH1* gene. This genetic change arises from cytosine to thymine substitution at position 350 in the nucleotide sequence (25, 26), causing codon 117 undergoes a pathogenic transformation, replacing a polar threonine with a hydrophobic methionine in a highly conserved domain of the *MLH1* gene.

Different tests are used for the detection of LS associated with CRC, such as colonoscopy, genetic testing, MSI, immunohistochemistry (IHC) and others. Immunohistochemistry, used for screening patients with suspected LS, can detect approximately 74% of MLH1 germline mutations when using MLH1-specific antibodies (27). This test uses antibodies against four MMR proteins (MLH1, MS2, MSH6, and PMS2) to assess their expression in colorectal cancer tissue (28). For example, the biological interpretation of the *MLH1* gene mutation could be shown by IHC that has demonstrated a reduction in DNA repair activity in cells carrying the Thr117Met variant which is associated with increased MSI. Functional analyses reveal reduced protein expression and compromised mismatch repair (MMR) activity linked to the disruption caused by the Thr117Met variant (29, 30). Moreover, this missense mutation likely impairs mismatch repair function by affecting the mutant protein’s ATP binding and hydrolysis, interfering with the formation of protein complex like hPMS2.

Considering that individual A has adenocarcinoma of the colon and carries a mutation in the *MLH1* gene, MSI analysis of the tumor samples is recommended. This test is essential not only for confirming the diagnosis of LS but also for its prognosis, as MSI has been established as a key biomarker in CRCs. In the general population, the rate of MSI varies between 12 and 17% but has been reported to be as high as 27 to 35% in patients younger than 30 years (31). In the case of 26-year-old A, an evaluation of MSI would not only contribute to a better understanding of his diagnosis but is also essential to guide appropriate follow-up and treatment for both him and his brother.

Determination of MSI has important prognostic value, as tumors with high instability are often associated with a better clinical prognosis. In addition, MSI has been established as a predictive biomarker in the context of immunotherapy, suggesting that immune checkpoint inhibitors may be more effective in these cases. This is because tumors with a higher mutational burden, characteristic of elevated MSI, generate a greater number of neoantigens (32). These neoantigens can stimulate an immune response, making MMR-deficient tumors particularly responsive to immune checkpoint inhibitors, such as anti-PD-1/PD-L1 therapies (33–35). In our case, the MLH1 c.350C > T variant, associated with increased microsatellite instability, could potentially create a pro-inflammatory tumor microenvironment. Understanding the interplay between mismatch repair gene mutations and immune response could lead to personalized immunotherapeutic strategies for Lynch Syndrome patients (14, 15). In this context, performing MSI analysis is not only crucial for the diagnosis and treatment of individual A, but also for the preventive management and follow-up of individual B, given the possible heritable involvement of mutations in the MLH1 gene (36).

This study marks the first report of this variant in Ecuadorian patients. Nevertheless, the variant has also been previously identified in unrelated families from Uruguay and Argentina, suggesting that the c.350C > T variant may be present within the broader Latin American gene pool (37). Notably, the reported frequency of the alternative allele T is 0,000 in the Latin American population (38).

Furthermore, this variant was identified in other populations. For instance, Yanus et al. (39) studied Russian individuals with either colorectal or endometrial cancer, who were referred for LS testing, revealing the *MLH1* c.350C > T variant. The results are consistent with the UMD-MMR database, which includes 55 entries for the mutation in French laboratories until 2015 (40). Moreover, another study focused on analyzing the *MLH1* gene in Polish and Baltic State families, revealing the mutation in one family with colon cancer (19). Another study reported the mutation in a hereditary nonpolyposis colorectal cancer patient in Slovakia (26). These studies collectively highlight the recurrent nature of the mutation across diverse geographical regions. Despite its global prevalence, the mutation has not been previously reported in Ecuador (14).

In the tumor sample from individual A, a *PTEN* c.968dup variant was identified. This variant results in a frameshift and a premature stop codon, probably producing absent or truncated PTEN protein. The truncation caused by c.968dup disrupts critical domains such as C2, C-tail, and PDZ, which play pivotal roles in protein localization, stability, and interaction with other proteins (41, 42).

*PTEN* is a tumor suppressor that regulates the PI3K/Akt signaling pathway, which is crucial for cell growth and survival. In colorectal cancer, loss of *PTEN* function leads to increased activity in this pathway, promoting tumorigenesis (43, 44). Other research suggests that loss of PTEN in CRC is closely associated with increased genomic instability and worse clinical outcomes, such as more advanced disease stages and the development of liver metastasis (45, 46). In this case study, the pathogenic variants in the *PTEN* and *MLH1* genes identified in individual A could trigger an additive effect from mutations in different genes, leading to a more aggressive disease phenotype.

However, this relationship is not consistently observed across all studies, suggesting that the role of *PTEN* in tumor progression may be influenced by other genetic or epigenetic factors (45–48). Moreover, in an extensive study involving 1,093 cases of CRC, *PTEN* mutations were detected in 43 tumors. Among these mutations, 47.3% were identified as nonsense mutations, 38.2% as frameshift mutations, 9.1% as missense mutations, and 5.5% as splice site mutations. Notably, the c.968dup variant was among the frameshift mutations closely related to CRC (49). This variability highlights the molecular complexity of CRC, particularly in cases linked to LS, and underscores the importance of further investigation to fully understand the impact of *PTEN* on CRC development and progression.

On the other hand, individual B carries a *FH* c.580G > A mutation, which has been implicated in hereditary cancer predisposition (50). The variant frequency has not been reported among the Latin American population. The c.580G > A variant, also denoted as p.Ala194Thr, is situated within the coding sequence of exon 5 in the *FH* gene. This genetic alteration arises from the substitution of alanine for threonine at codon 194. Notably, alanine is characterized as neutral and non-polar, whereas threonine possesses a neutral and polar nature. Consequently, advanced protein modeling techniques, coupled with the scrutiny of their physical properties, have revealed that this missense variant disrupts the functionality of FH protein (51). Despite this, the variant has been categorized as a variant of uncertain significance, primarily due to findings from RNA analysis suggesting that its impact on mRNA splicing does not significantly alter splicing patterns. Furthermore, associations have been made between this variant and other types of cancer such as pheochromocytoma, paraganglioma, and renal cell cancer. However, its low population frequency (0.00007), mainly present in individuals of European descent, makes it uninformative for assessing its pathogenicity (51, 52).

Furthermore, the PTEN protein has a physical or functional interaction with MLH1, while the FH protein does not show any interaction with *PTEN* or MLH1 (Supplementary Figure S2). However, the coexistence of these variants in individuals could have synergistic effects, potentially increasing their cancer risk. Even though the assay employed in this investigation covered some genes implicated in mismatch repair, it would be interesting to utilize a multigene panel encompassing an extensive spectrum of genes or SNPs associated with LS.

The genetic analysis of the ancestral components of the two brothers harboring the *MLH1* c.350C > T variant, along with additional variants (*PTEN* c.968dup for individual A and *FH* c.580G > A for individual B), has provided a further understanding of the possible relationship between genetic ancestry and specific mutations associated with hereditary cancer predisposition.

Moreover, the ancestral composition of the individuals revealed a mosaic of African, European, and Native American components, as previously reported for the Ecuadorian (14). However, both siblings have high levels of Native American ancestry, which is notable given a lack of data on population specific genetic risks in this group. Although ancestry data does not directly elucidate cancer predisposition in this case, the Native American heritage may offer insights into underrepresented genetic variants relevant to Lynch Syndrome and cancer. Further studies on larger cohorts are needed to explore the relationship between ancestry and cancer risk in Latin American (14).

The *MLH1* c.350C > T variant, associated with Lynch syndrome, has been extensively studied and reported in different regions, such as Latin America (37) and Europe (19, 26, 39, 40). Besides, a study compared the racial differences in *MLH1* mutations between the Caucasian and East Asian races and found that this variant ranks among the top ten mutations in both ethnic groups (53). The prevalence of this mutation in individuals with predominantly Native American ancestry is of particular interest and has not been previously reported, which could provide insights into population-specific genetic risk factors. However, the *PTEN* and *FH* variants have not been previously described in other populations.

Consequently, employing multiple gene sequencing may benefit patients, especially those with personal or familial histories. The identification of individuals with hereditary cancer predispositions, such as Lynch syndrome, holds significant promise for reducing cancer incidence and mortality rates. Nonetheless, accessing comprehensive mutation screening may present challenges in certain scenarios. However, once mutations are identified, screening other family members for the same mutation can be achieved using relatively straightforward methods (15).

In this study, we used NGS panels that included the MMR genes (*MLH1, MSH2, MSH6, or PMS2*), identifying a pathogenic *MLH1* mutation in both siblings. This finding underscores the importance of genomic testing in detecting germline mutations within families. Clinically, it enables a more precise diagnosis and treatment for Individual A, who is symptomatic, while providing necessary follow-up for Individual B, who remains asymptomatic.

A limitation of this study is the budget constraint, because NGS could not be performed on all family members. Despite this constraint, the screening process remains informative and contributes significantly to continuous patient monitoring. Consequently, the adoption of massive sequencing approaches may provide valuable insights, enabling the implementation of effective cancer prevention strategies, personalized screening protocols, and tailored therapeutic interventions to mitigate cancer-related morbidity and mortality linked to Lynch Syndrome (11, 54).

In conclusion, the ancestral components, and associated variants of the two siblings provide the first data of a mainly Native American composition and mutations related to LS and cancer predisposition. Further studies on diverse populations and larger cohorts could improve the understanding of population-specific variants, their implications, and healthcare strategies.

## Data Availability

The original contributions presented in the study are included in the article/Supplementary material, further inquiries can be directed to the corresponding author/s.

## Supplementary material

SUPPLEMENTARY FIGURE S1 Ancestral composition of the subjects under analysis (in yellow).

SUPPLEMENTARY FIGURE S2 Protein – protein physical and functional association (constructed with STRING).
