# Supplementary figures and images for: Case report: Exploring Lynch Syndrome through genomic analysis in a mestizo Ecuadorian patient and his brother
Source: Front Med (Lausanne). 2024 Dec 17;11:1498290. doi: 10.3389/fmed.2024.1498290 (PMC11685006; doi:10.3389/fmed.2024.1498290)

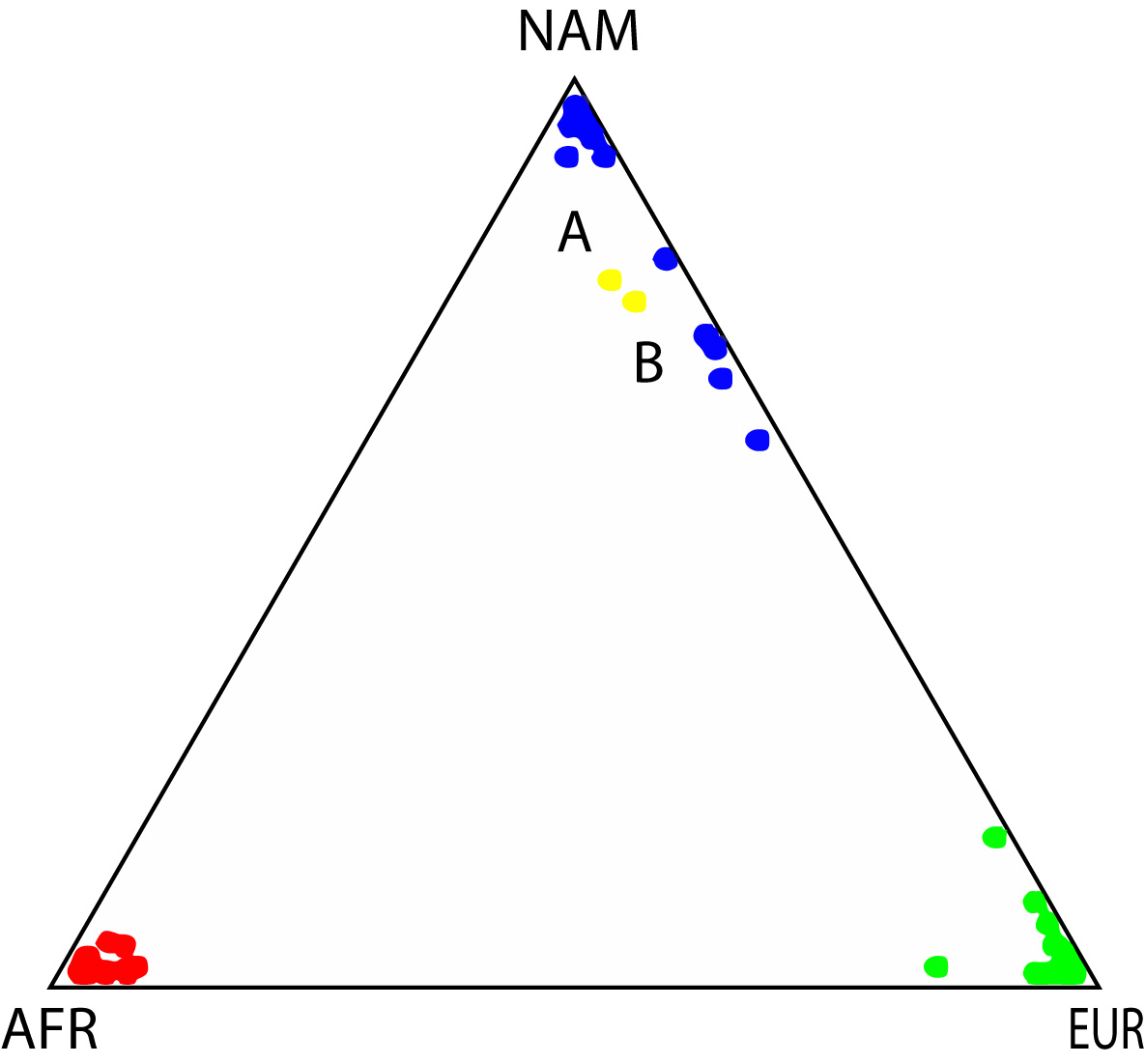

Supplement: SUPPLEMENTARY FIGURE S1 — Ancestral composition of the subjects under analysis (in yellow). [file Image_1.JPEG]

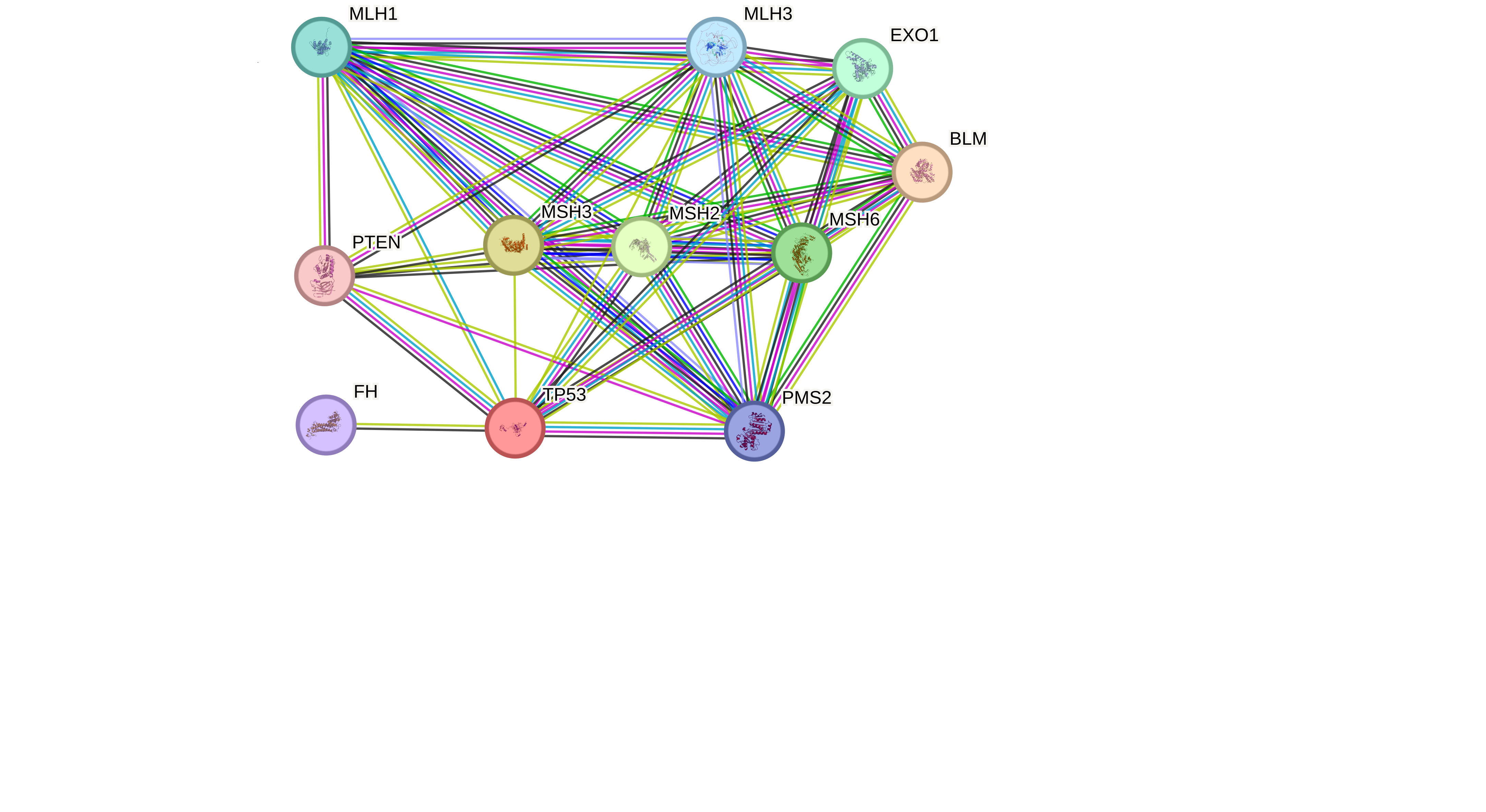

Supplement: SUPPLEMENTARY FIGURE S2 — Protein – protein physical and functional association (constructed with STRING). [file Image_2.TIFF]
